# The influence of climate change on the sesame yield *in North Gondar, North Ethiopia*: Application Autoregressive Distributed Lag (ARDL) time series model

**DOI:** 10.1186/s12870-024-05203-4

**Published:** 2024-06-06

**Authors:** Dagnew Melake Abebe, Dagne Tesfaye Mengistie, Aychew Alemie Mekonen

**Affiliations:** Department of Statistics, College of Natural and Computational Science, Jigjiga University, Jigjiga, Ethiopia

**Keywords:** Yields of sesame, Rainfall, Temperature, ARDL, ECM, Co-integration and FE

## Abstract

Sesame is a major annual oil crop that is grown practically everywhere in tropical and subtropical Asia, as well as Africa, for its very nutritious and tasty seeds. Rising temperatures, droughts, floods, desertification, and weather all have a significant impact on agricultural production, particularly in developing countries like Ethiopia. Therefore, the main objective of this study is to examine the influence of climate change on the sesame yield in North Gondar, North Ethiopia, by using the autoregressive distributed Lag (ARDL) time series model. This study employed climate data from the Bahirdar Agrometeorological Center and secondary data on sesame production from the Ethiopian Statistical Service, spanning 36 years, from 1987 to 2023. Autoregressive Distributed LAG (ARDL) includes diagnostic tests for both short- and long-term autoregressive models. The results for the long-run and short-run elastic coefficients show a significant positive association between temperatures and sesame yield. Sesame yield and rainfall have a significant negative long-run and short-run relationship in North Gondar, North Ethiopia. ARDL results confirm that temperature and rainfall have significant effects on sesame productivity. Temperature had a considerable favorable effect on sesamen production, but rainfall had a negative effect in North Gondar, Ethiopia. Based on the evidence acquired from our study, we made several policy recommendations and suggestions to government officials, policymakers, new technologies, researchers, policy development planners, and other stakeholders in order to develop or implement new technology to halt its production and direct adaptation measures in light of the certainty of global warming and the characteristics of climate-dependent agricultural production.

## Introduction

Global weather and climate-related phenomena, including drought, flash floods, unexpected rain, frost, hail, and storms, account for the majority of annual crop losses in agriculture. Drought, flooding, desertification, rising temperatures, and other weather extremes have a negative impact on agriculture, particularly in developing nations [1]. According to reports on climate change, small-scale irrigation management, which accounts for 12.75 percent of farms in Ethiopia, is a crucial tactic used by sesame producers to temper the effects of the phenomenon. Another tactic they use to adapt to climate change is the adoption of sesame as their primary crop diversification method [2].

Climate change and variability may also have some positive effects that can be used to improve food security, as evidenced by the impact of these factors on the occurrence of sesame phyllody and the disease's symptomatology. As such, these cases should be taken into account in crop management plans and strategies as well as modeling studies that attempt to predict the effects of climate variability [3]. The lack of water in dry regions, which typically occurs during hot weather, makes it critical to have a useful decision-support tool for evaluating irrigation management techniques, strategies, and practices [4].

The higher frequency and intensity of extreme weather events, such as temperature and rainfall, are predicted to have a severe impact on agriculture in many locations [5]. Climate change is expected to have a more severe effect on low-income residents of rural agricultural communities in developing nations, the most of which are found in dry regions. The biggest danger to these regions' agricultural, food security, and sustainable development is climate change [6, 7]. The primary cause of the consistently falling amount of sesame production in the country's sesame growing areas is climate variables, specifically the lack of water and high temperatures that occur in the middle of summer. According to reports, the two biggest issues affecting the production of numerous crops, including sesame, are drought and inadequate irrigation water [8, 9].

Sesame seeds from Ethiopia are in great demand outside, and some research suggests that there is still room for the crop to be grown on productive land throughout the nation [10]. Sesame production in 2017–2020 was predicted to cover 171,417 hectares with a yield of 65,207 tons, mostly from three regions (East Wellega, Western Tigray, and North Gondar), according to data released by the Ethiopian Export Promotion Agency [11] in 2014 [12]. Oromia, Benshangul Gumuz, Amhara, and Tigray are Ethiopia's principal sesame-producing regions [13].

Sesame led the oil crop production rankings for the 2017–18 cropping season [14]. Ethiopia's main sesame-producing regions are Tigray, Amhara, Oromia, and Benshangul Gumuz [15, 16]. Sesame is a significant and profitable commodity crop that is farmed and exported from the Amhara Region. In terms of production and area covered by sesame, North Gonder in the Amhara region placed first (33.6%) and second (31.8%), respectively, in the results of the CSA Agricultural Sample Enumeration Survey (2017/2020). At 6.85 quintals, the region's productivity score puts it ahead of all other regions [17, 18].

Ethiopia has the most variable rainfall pattern. Several individuals and organizations have published scientifically fascinating studies on the variability of Ethiopian rainfall, among other things, by dividing Ethiopia into distinct temporal and spatial rainfall categories. As a result, a drought occurs in northern Ethiopia every three to six years, and in other parts of the nation every seven to eight years [19]. The onset of the rainy season is another source of concern in Ethiopia. It used to start in March but now moves to April and ends in July. As a result, the growing seasons and seasonal rainfall totals are becoming shorter [20].

Ethiopia is a rainfall-dependent agricultural country that is vulnerable to the effects of climate change and risks.Floods and droughts, which are increasingly frequent and extreme, have a major and negative impact on Sesame production [21]. According to the trend in sesame output, there were 400,000 hectares of sesame production in 2016. By 2019, the area under sesame farming had dropped to less than 2000 ha [21]. According to [22], the primary reason for the decrease in production is that large-scale sesame production areas have dramatically decreased as state and private farms specializing in this crop have become inoperable or operating at a minimum scale, owing primarily to a lack of labor at harvest, climate change, drought, and other management and social problems, particularly in 2010. However, due to a variety of biotic and abiotic conditions, sesame productivity is declining year after year [23].

Sesame yield is undeniably important to Ethiopia's economic well-being, employing more than 80% of the population and contributing for 50% of GDP [24]. While the country possesses around 3.7 million hectares of irrigable land and 110 billion cubic meters of surface water, the cropping system is primarily based on rainfed circumstances [25]. Ethiopia's agricultural economy is subsistence-oriented, which means it produces enough food to endure only from one harvest to the next. As a result, failure of one harvest meant malnutrition for the following year, a scarcity of seed for the next cropping season, and a loss of animal strength to plow the land [26]. The climate of Ethiopia's arid and semi-arid regions is marked by significant rainfall variability, unpredictability, strong winds, high temperatures, and high evapotranspiration [27]. It is so critical to evaluate its effects, particularly on sesame yields, because it is most likely to be impacted by rapid or gradual adverse changes in climatic circumstances. One of the most significant issues confronting emerging countries like Ethiopia today is establishing national food security and diversifying export-earning agricultural commodities.

This study further varies from earlier studies in that it dynamically combines rainfall, temperature, and agricultural yield using the ARDL model, a well-established time series technique that incorporates short- and long-term tests. As far as we know, the ARDL model has never been used to study the impact of climate change on sesame yield in Ethiopia, instead relying on ANOVA, Moving Average (ARMA), and Vector Auto-Regressive (VAR).The primary goal of this study is to investigate the impact of climatic change on sesame yield in North Gondar, North Ethiopia, using the autoregressive distributed Lag (ARDL) time series model.

## Literature review

### Theoretical review

Sesame produced in certain locations of Ethiopia receives approximately 300–700 mm of rainfall each year [5]. Ethiopia's largest sesame-producing regions are located in the lowlands of the North and South West [28]. In addition, the country produces a wide range of sesame seeds, including the Humera, Gondar, and Wollega varieties, which are widely known in international markets. Humera and Gondar sesame seeds are ideal for bakery and confectionary applications due to their white appearance, sweet flavor, and scent. On the other hand, Wollega sesame has a high oil content, giving it a significant competitive edge in edible oil manufacturing [29].

Sesame has a very broad root system, which contributes to its drought tolerance. However, enough moisture is required for germination and early development. For optimal sesame yields, a minimum of 300–400 mm of rainfall is required per season [30]. Moisture levels have the biggest impact on production prior to planting and flowering. Sesame tolerates waterlogging. Late-season rains extends growth and increases shattering losses. Wind can induce shattering during harvest, which is mentioned as one reason for the commercial failure of sesame cultivation. Sesame is photoperiod sensitive; it is a short-day plant whose flowering begins with day-length shortening and continues until it reaches a critical level, which varies by variety. The oil content of the seed tends to increase with increased photoperiod. Because protein content and oil content are inversely proportional, seeds with increased oil content have decreased protein content [31].

Climate change has affected many regions of the world in recent years, and its impacts are predicted to worsen in the future decades. Climate change is one of the phenomena that the globe is currently witnessing. Agriculture and natural resources rely heavily on weather, therefore climate variability and changes, both short-term (during the growing season) and long-term, play a critical role in their production and sustainability [32]. The spatial–temporal dynamics of meteorological variables in the context of climate change, particularly in countries where rain-fed agriculture is prevalent, are critical for assessing climate change and proposing viable adaptation solutions [33]. Climatic variabilities include the types of changes (temperature, rainfall, and the occurrence of extremes); the magnitude and rate of climate change that affects public health, agriculture, food security, forest hydrology, water resources, coastal areas, biodiversity, human settlement, energy, industry, and financial services; and changes in physical and socioeconomic systems that have been identified in many regions [34].

### Empirical review

Climatic unpredictability has a significant impact on several economic sectors, including agriculture, forestry, water resource management, road maintenance, construction, tourism, and public transportation [35]. sesame is the most extensively cited human activity that is expected to be affected by climate change [36]. Climate is the key driving element behind sesameproductivity [37]. Climate influences a wide range of agricultural activities, outputs, and input resources, including yields, land quality, on-farm storage, water supply, labor migration rates in urban and rural communities, population growth, farm revenue, and farmer skills [38].Climatic fluctuations have a wide-ranging impact on sesame agriculture, affecting all aspects of production management, from seedbed preparation to harvesting [39].

The study investigates the impact of climatic changes on sesame production in South Africa and across much of Africa. To assess the general impact of climate change on sesame, they look at rainfall and temperature data for South Africa's nine provinces from 1970 to 2006. The relationship between rainfall, temperature and sesame yield were examined using ANOVA software, and the covariance of rainfall and temperature was very significantly negative in most provinces. Except in arid places, the covariance between temperature and rainfall has increased during the previous decade. As the temperature rises, rainfall decreases in all places [40].

Attempts to demonstrate rainfall sesame yield patterns and provide insight into the development of an early warning system in Ethiopia using time series analytic techniques. Rainfall fluctuations and sesame yield responses to rainfall, as well as past yield shocks, are analyzed using Auto-Regressive Moving Average (ARMA) and Vector Auto-Regressive (VAR). According to the results of the VAR estimation, current levels of sesame yield respond to historical levels of yield even more than to rainfall in most areas [41].

## Method and material

### Description of the study area

**Fig. 1:**
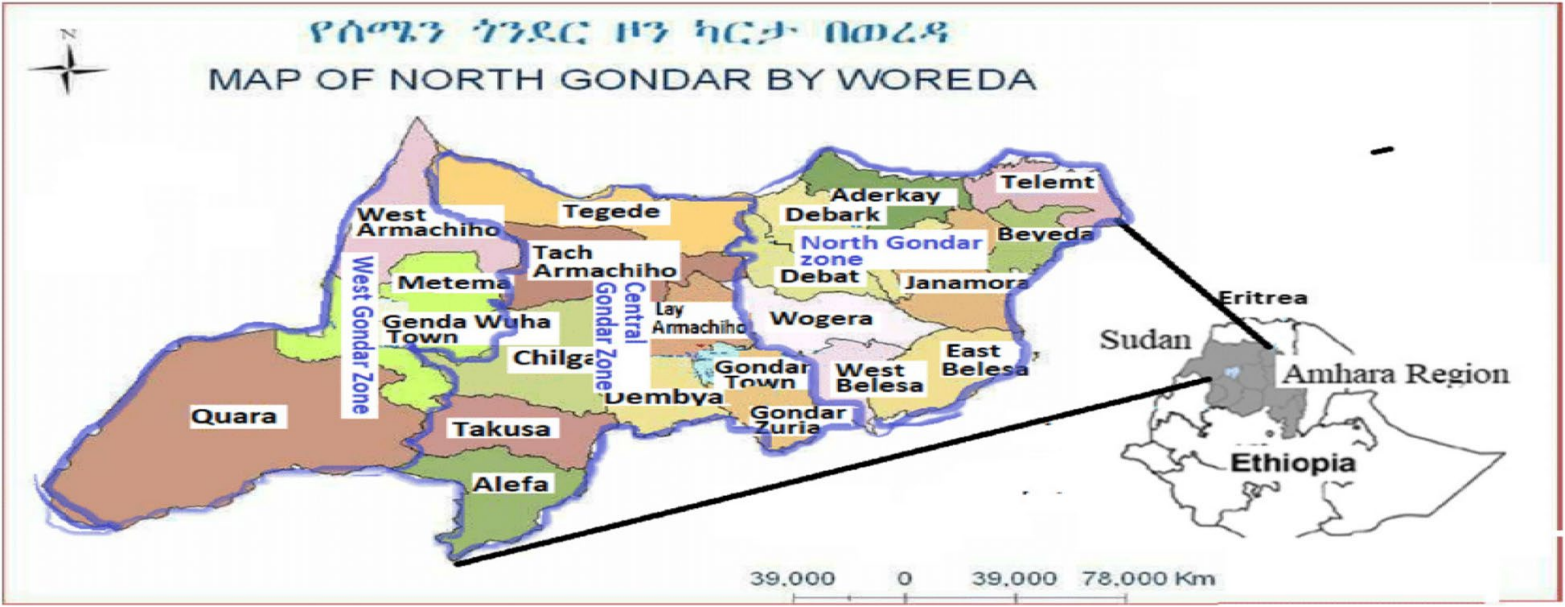
Map of North Gondar Zone by wereda

North Gondar Zone is located at 1203'N, 37,028'E. Gondar is 727 km from Addis Ababa, Ethiopia's federal capital, and 120 km from Bahir Dar, the capital of Amhara National Regional State. Gondar's entire size is 192.3 km2, with undulating mountainous terrain [42].North Gondar Zone, one of the 11 administrative zones that make up Ethiopia's Amhara National Regional State (ANRS), is the location of this study.North Gondar shares borders with the Tigray Region to the north, the Central Gondar Zone to the south, and Wag Hemra to the east. North Gondar is home to the towns and cities of Arbaya, Dabat, Dembiya, Debark, Emfranz, Feres Megria, Musebamb Town, Kurbi, Armachiho, Gondar, Tekeldengy, Gorgora, and Metemma [43, 44]. Bega is the dry season, whereas Kiremt and Belg are the zone's major and minor wet seasons. The primary causes of the seasonal shifting of the Inter Tropical Convergence Zone (ITCZ), which moves northward in July and southward in January, and the complicated topography with a discernible altitude difference are to blame for the variability of rainfall in terms of its beginning and ending dates, amount, and distribution. Because of climate change, the rainy seasons (Kiremt and Belg) are becoming more inconsistent [45]. Figure 1. Map of North Gondar Zone by wereda Fig. 1. Map of North Gondar Zone by wereda Fig 1. Map of North Gondar Zone by wereda

### Data source and collection

This study was used of secondary data from the Ethiopian Statistics Service (ESS) and the Bahirdar Agrometeorological Center. Data on sesame yield for the years 1987–2023 was obtained from the Ethiopian Statistics Service.Sesame production is valued annually in quintals per hectare. The Bahirdar Agrometeorological Center provided the temperature and rainfall data.The basis for this study was the 36 consecutive summer seasons (Jun, July, August, and September) yearly time series data that were observed between 1987 and 2023 GC. The annual summer rainfall in millimeters (mm), the average temperature in degrees Celsius (°C), and the matured variety of sesame yield in North Gondar, expressed in quintals per hectare (Qt/hat), serve as control variables in the research region see Table 1.

**Table 1:** Decription of variable and data source

Variable	Decription	Unit
Tem	Temprature	Measured in degree Celsius (^0^c)
Sesamen	Sesamen yield	Measured in quintals per hectare (Qt/hat)
Rain	Rainfall	Measured in milli meters (mm)
Tim	Time lag	-

Source: ESS-Ethiopaina Statistics Service and the Bahirdar Agrometeorological Center from 1987 and 2023 GC

### Data quality control assessment

Before starting the analysis, validity checks (quality control) were performed for the historical time series climate data. Errors arising from data digitalization, reporting, and internal data inconsistencies, such as minimum temperature exceeding maximum temperature and rainfall values less than zero (0 mm), were assessed and handled in this study [45]. Additionally, in order to determine if the values in a time series data are indeed outliers or just naturally extreme values, outliers were carefully detected. The outliers were managed using a typical outlier threshold, which is defined using the inter-quartile range (IQR) [46].The following formula was used to estimate the threshold values:

$$\mathrm{threshold}=(\mathrm Q1-3\mathrm{IQR},\mathrm Q3+3\mathrm{IQR})$$

In this case, the first quartile is denoted by Q1, the third by Q3, and the inter-quartile range (IQR) is the difference between Q3 and Q1. The inter-quartile range approach is renowned for its ability to withstand outliers while retaining extreme data. After that, the identified outlier values were eliminated and replaced with the outlier threshold.

### Analytical techniques

The statistical methodology presented currently employs the Statistical Package for Social Science (SPSS Version 23) and EViews 13 to estimate short-term, long-term, and ARDL correlations between temperature, rainfall, time lag, and sesame production.

#### Summary statistics

Before proceeding with statistical analysis, an initial study is required to show the time series components. Sesame yield and meteorological factors are analyzed using basic descriptive statistics such as maximum, minimum, mean, standard deviation (S.D.), skewness, kurtosis, and the Jarque–Bera normality test. Positive and negative connections are determined by examining the correlation between variables.

#### Stationarity test

Unit root tests are used in many statistical processes and models to determine whether or not elements of a time series are stable. To be called stationary, a time series must preserve its basic statistical features over a long period. An unexpectedly ordered pattern or unit root in a chronology. The Dickey-Fuller, Augmented Dickey-Fuller, and Phillips-Perron tests for unit root are all relevant, but the Augmented Dickey-Fuller test is used here because it is the most robust [47].

#### Information criteria selection

The criterion with the lowest value indicates the ideal lag time to apply since it ensures model stability [48]. The goal of selecting the right lag is to lower the residual correlation information criterion utilized in the widely used model selection [49]. The information criteria utilized to calculate the appropriate lag order using the ARDL approach are LR, FPC, AIC, SIC, and HQC, assuming serially uncorrelated residuals [50].

#### Cointegration test

After working with several equations, the Johansen cointegration test is used. This A test was conducted to discover if or not a long-term relationship existed. The Trace method of maximum likelihood was used.

#### Auto Regressive Distributed Lag (ARDL) Model

Due to the inaccuracy of the existing unit root test, the ARDL strategy was developed to avoid these prerequisites and directly determine the integration order of the variables. When examining the hypothesis of long-run and short-run coefficients of input variables, ARDL was shown to be resilient regardless of whether they are integrated at mixed levels, viz., I (0) and I (1). Model selection in the ARDL technique was based on the Akaike Information Criterion [49]. The ARDL model is represented as follows in the Unrestricted Error Correction Model (UECM).

$$\Delta DYL = {\gamma }_{0}+ {\sum }_{\text{i}=1}^{\text{p}}{\upbeta }_{1\text{i}}\Delta {\text{DYL}}_{\text{t}-\text{i}} + {\sum }_{i=1}^{p}{\beta }_{2i}\Delta {DTM}_{t-i}+ {\sum }_{i=1}^{p}{\beta }_{3i}\Delta {DR\text{F}}_{t-i} + +{\Theta }_{1}{DYL}_{t-i} +{\Theta }_{2}{DTM}_{t-i}+{\Theta }_{3}{DR\text{F}}_{t-i}+ {U}_{1t}$$

where DYL is the first lagged value of annual sesame yield at time t, DTM and DRF are the first lagged values of Sameer (cremt) seasonal average temperature and rainfall, respectively. The bounds test is mainly based on the joint Wald test or F-test, whose asymptotic distribution is non-standard under the null hypothesis of ‘no cointegration. If the calculated F statistic is between the upper and lower bound critical values, the inference is inconclusive, and we need to know the order of integration of the underling variables before we make a conclusive inference [51].

#### Diagnostics test for ARDL model

Pesaran [52] demonstrated that a model can only be considered accurate if it meets all of the CLRM assumptions. The ARDL model's most crucial diagnostic assumption is that the endogenous variable's residuals are independent [53]. Homoscedasticity implies that the residual variability in the response variable remains constant, hence there is no heteroscedasticity. This study examines various assumptions, such as no heteroscedasticity, serial correlation, and response variable normality, to see how near this model is to the actual model for making accurate and valid judgments. CUSUM and CUSUMSQ tests are used to evaluate the stability of a generated model [54].

#### Forecasting and measures of forecasting accuracy

Forecasting is the ultimate aim of ARDL and *M* estimation. Since multivariate time series analysis is a continuation of univariate time series analysis, the forecasting process is nearthe sameame in boscenarios. os.A forecasting horizon for ℎ ≥ 1 of an empirical (*p*) process can be generated in this work. Accuracy measurement studies are regarded as a prerequisite before beginning a forecasting project. Thiel's U statistmeanMperceivedrerrorrror (mean), meanMperceivedrabsolute errorrror (MPAE) were all differently calculated.

## Result and discussion

### Summary statistics

Table 2 showed that the average sesame yield (Qt/hat) during the study period was 4.79, with a minimum (2.12), maximum (6.92), skewness (-0.14), kurtosis (1.62), and Jarque–Bera test indicating a normal distribution. Because all of the Jarque–Bera statistic's *P*-values are greater than 5%, the null hypothesis of a normal distribution for all variables is not rejected at the 5% level of significance. The temperature rose from 18.21 °C in 2001 to 29.12 °C in 2018, but rainfall declined from 300.56 mm in 1998 to 180.9 mm in 2017.

**Table 2:** Summary of descriptive statistics

Descriptive Statistics	Yield(Qt/hat)	Temperature(${C}^{0}$)	Rainfall(mm)
Mean	4.79	24.03	243.77
Median	4.82	24.32	252.54
Maximum	6.92	29.12	300.56
Minimum	2.12	18.21	180.65
Standard deviation	1.52	2.79	42.37
Skewness	-0.14	-0.09	-0.13
Kurtosis	1.62	2.11	1.52
Jarque–Bera	2.96(0.223)	1.24(0.54)	3.37(0.19)
Observation	36	36	36

### Test of stationary nature of the data

#### Unit root test

**Fig. 2:**
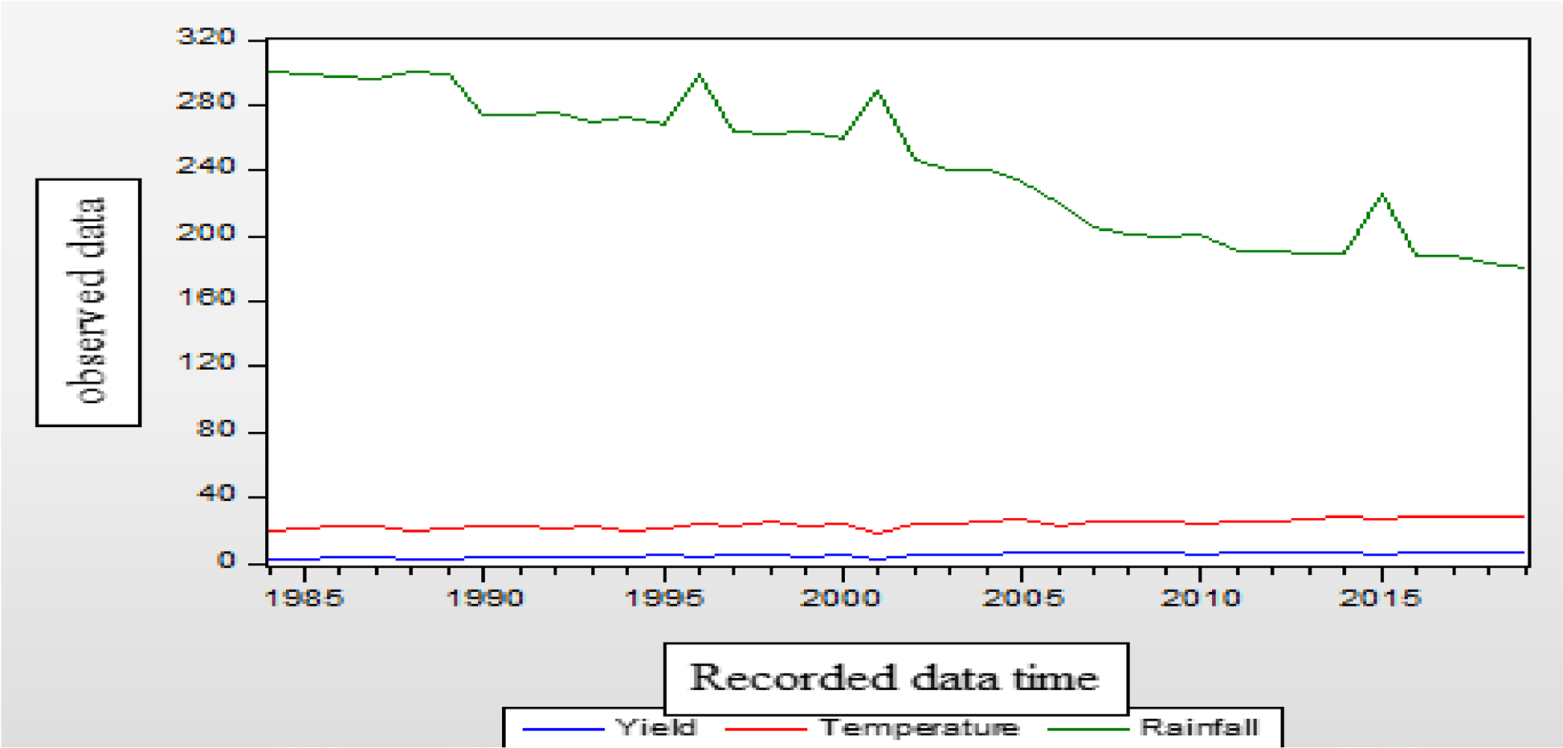
Time series plot of rainfall, temperature, and yield (at level)

**Fig. 3:**
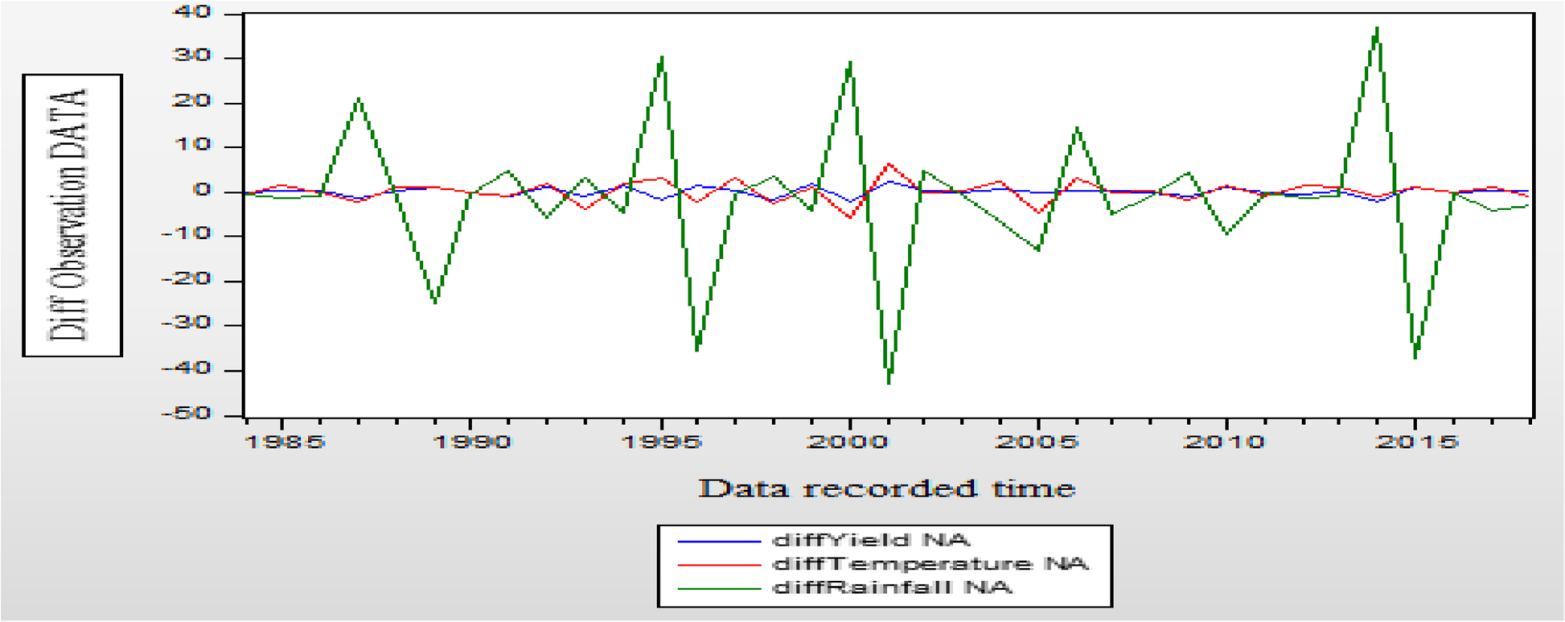
Time series plot of yield, rainfall, and temperature (after first deference)

If a unit root is present, the time series under consideration becomes non-stationary, as seen in Fig. 2. It shows that temperature and sesame yield are trending upward. Nonetheless, given the graph's up-and-down fluctuation pattern, the rainfall indicates some unpredictability. This indicates that none of the variables are stationary and that the data mean has fluctuated. Figure 3 does not state that all variables are strictly stationary, but rather that each variable must exhibit stationary time series behavior after the initial difference in order to fit the time series model.

#### ADF and PP unit root test for stationary at level

The findings of ADF and PP show that yield, temperature, and rainfall are non-stationary, as shown in Table 3. The test also detected a unit root at the level, both with and without a trend. The unit root null hypothesis is rejected for the initial discrepancies between the three variables with and without intercept and trend. This implies that the three variables in the time series yield, temperature, and rainfall are integrated to degree one (or order one). As a result, all variables are non-stationary at levels but stationary at initial differences, according to the findings of the ADF and PP tests in Tables 3 and 4. It is advantageous to adopt the ARDL model when all variables are stationary at the first difference as can be inferred from Table 3. Therefore, the best approach for estimating or verifying the long-term relationship between the research variables is the ARDL cointegration methodology.

**Table 3:** ADF and PP unit root test for stationary at level

SERIES	Level with Intercept	Level with Intercept and the trend	Level without Intercept and trend
	Test statistic ADF PP	Test statistic ADF PP	Test statistic ADF PP
Yield	-1.21[0.659] -2.03[0.272]	-2.84[0.196] -3.32[0.521]	1.47[0.962] -0.11[0.640]
Temperature	1.03[0.996] 2.54[0.114]	-3.02[0.146] -3.12[0.051]	-2.74[0.997] -1.88[0.983]
Rainfall	-0.55[0.869] -0.52[0.871]	-3.95[0.058] -3.95[0.059]	-2.02[0.054] -2.66[0.059]
1% critical value	-3.66 -3.63	-4.32 -4.24	-2.83 -2.63
5% critical value	-2.96 -2.95	-4.01 -3.99	-2.95 -1.95
10% critical value	-2.62 -2.61	-3.98 -3.97	-1.61 -1.62
Conclusion	Non-stationary	Non-stationary	Non-stationary

**Table 4:** ADF and PP unit root test for stationary at first difference

SERIES	1st difference with Intercept	1st difference with Intercept and Trend	1st difference wit out Intercept and trend
	Test statistic ADF PP	Test statistic ADF PP	Test statistic ADF PP
Yield	-4.39[0.0016] -5.63[0.000]	-4.44[0.007] -5.77[0.000]	-6.12[0.000] -7.84[0.000]
Temperature	-3.11[0.03] -3.37[0.000]	-3.95[0.006] -4.78[0.000]	-6.12[0.000] -5.63[0.000]
Rainfall	-9.13[0.000] -10.69[0.000]	-8.99[0.000]-10.57[0.000]	-8.56[0.000] -8.56[0.000]
1% critical value	-2.87 -2.95	-3.11 -3.01	-2.63 -2.63
5% critical value	-2.61 -2.78	-3.55 -3.21	-1.95 -1.65
10% critical value	-1.55 -1.85	-3.12 -3.35	-1.66 -1.61
Conclusion	Stationary	Stationary	Stationary

### ARDL model specification

#### Bounds test for co-integration

There is a long-term association between the explanatory factors (rainfall and temperature) and the dependent variable (sesame yield). The explanatory variables of rainfall and temperature have a short-term relationship with the dependent variable of sesame production. As a result, draw the conclusion that the variables under examination have a cointegrating relationship (see Table 5).

**Table 5:** The F-statistic test

F-statistics	Lag Length	Significance	Lower Bound value I(0)	Upper Bound value I(1)
5.38	1	1 Percent	4.13	5
		5 Percent	3.1	3.87
		10 Percent	2.63	3.35

#### Autoregressive Distributed Lags (ARDL) model analysis

##### Determination order of lags for ARDL model

Based on Table 6, the AIC, SBIC, HQIC, FPE, and LR tests indicate that the appropriate lag length for the ARDL model is one (1), since the minimal AIC, SBIC, HQIC, and FPE values occur at lag one. As a result, it is reasonable to believe that the calculated ARDL model at lag one is the best fit for the data of all candidate models. The selection criteria of the AIC, SBIC, HQIC, FPE, and LR resulted in a lag order of one, and the author preceded further experiments with lags.

**Table 6:** Order specification for ARDL model test

Lag	Log	LR	FPE	AIC	HQIC	SBIC
0	-245.9064	NA	1144.907	15.55665	15.60220	15.69406
1	-207.7514	66.77127^a^	185.8190^a^	13.73446^a^	13.91665^a^	14.28411^a^
2	-201.4530	9.841205	223.9089	13.90331	14.22215	14.86520
3	-192.0084	12.98639	227.4141	13.87552	14.33101	15.24965
4	-812.0662	11.80627	233.2659	13.81664	14.40877	15.60301

^a^Indicates that the ARDL Order Selected by the Criterion

#### Lag exclusion test

Table 7 shows that initial lag is significant at the 5% level, meeting the ARDL model test's five requirements (Table 6). Otherwise, the value in square brackets is the probability value for the corresponding chi-square statistics. Lag one is significant for the response variable at the 5% level, as seen in Table 7. As a result, the chi-square test ensures that the ARDL model test at lag one is determined to be optimal for the data set and may thus be performed, as ARDL models normally require the same lag length for all series.

**Table 7:** ARDL lag exclusion wald test

Chi-squared test statistics for lag exclusion: Numbers in df are *p*-value		
Lag	Yield of sesame	Joint
Lag 1	35.49721[0.0000]	35.49721[0.0000]
Df	1	1

#### ARDL model parameter estimation

Table 8 shows that the limit and Wald tests of the F-Statistic value and Chi-square values are highly significant at a 5% level of rejection, implying a long-term link between the Sesame variable and the regressors. Because the results show that the null hypothesis's Wald tests of association assumptions (there is no long-run association between the response variable and the explanatory variables) are rejected, we conclude that there is a long-run association between the response variable and the explanatory variable.

**Table 8:** A long-run bound test using MLE test with Wald tests

Wald Test: Equation Untitled: Null Hypothesis: C(1) = C(2) = C(3) = C(4) = C(5) = 0 be coefficient of regressor for yield of sesame, Normalized Restriction(= 0)					
Test Statistics: F(5,29) 66.09710 [0.000], and CHSQ(5) 330.4855[0.000]					
Coefficient	C1	C2	C3	C4	C5
Value	0.166	-0.042	0.014	0.082	-0.11
Stan. error	0.187	0.006	0.010	0.053	0.053

C(1) the long-run coefficient of sesame yield (∆YL*t*-1), C(2)&C(3) are the long-run coefficient of rainfall(DRN and DRN*t*-1) respectively, C(4) and C(5) are the long-run coefficients of temperature (DTM and DTM_t-1_) respectively

#### Long-run ARDL model estimation

Table 9 indicates that the long-run estimates suggested that temperature (TM), lagged value of sesame yield ($\Delta$ YL_t-1_), and lagged value of rainfall (RN_t-1_) had a positive impact on the current production of sesame yield (YL) in the study area. The temperature coefficient indicates that the current temperature increase is greater than the productivity of sesame by eight percent and the short-run estimated coefficient of rainfall of -0.0424 indicates that a one percent increase in annual rainfall is greater than the productivity of sesame, which decreases by four percent. Sesame productivity is negatively impacted by total rainfall, according to the impulse response function of rainfall and sesame productivity. Sesame productivity is negatively affected by rainfall because sesame is a warm-season annual crop that is primarily adapted to areas with long growing seasons and well-drained soils. Sesame prefers slightly acidic to alkaline soils (pH 5–8) with moderate fertility. This study is in line with research conducted in Pakistan by [55] and the Ada'a district of the East Showa Zone of Oromia Regional State by [56], both of whom used the vector autoregressive model. This study contradicts the study in China, in which the increase in precipitation has a significant positive effect on sesame yields; however, the increase in temperature year by year has a significant negative effect on sesame yields [57].

**Table 9:** Estimated long run coefficients using the ARDL approach

Regressors	Coefficient	Std. Error	T-Statistics [Prob.]
$\Delta$YL(-1)	0.165917	0.186825	0.888088 [0.3818]
RN	-0.042421^a^	0.006009	-7.059732 [0.0000]
RN_t-1_	0.013652	0.010029	1.361272 [0.1839]
TM	0.081898^b^	0.053190	1.539746[0.01345]
TM_t-1_	-0.106468^c^	0.052946	-2.010873 [0.0537]
CONS	11.54726	3.724852	3.100058 [0.0043]

R-Squared = 0.919329 F-Statistic = 66.097 Adjusted R-Square = 0.905420 Prob (F-Statistic) = 0.000

^a,b,c^Indicates statistically significant at 1%,5% and 10% respectively. The dependent variable is DYL: Model selected ARDL(1,1,1), selected based on Akaike information criteria

$$\Delta\mathrm{YL}=11.547+0.166\Delta{YL}_{t-1}-0.042\mathrm{RN}+0.014{RN}_{t-1}+0.082\mathrm{TM}-0106{\mathrm{TM}}_{t-1}+{0.834ECT}_{t-1}$$

#### Short run error correction model

The error correction term (ECM) in Table 10 indicates the speed of adjustment to restore equilibrium in the dynamic model. It is a lagged period residual obtained from the estimated dynamic long-run model. The coefficient of the error correction term indicates how quickly variables converge to equilibrium. Moreover, it should have a negative sign and statistically significant at a standard significant level (i.e. *p*-value should be less than 0.05) show Table 10.

**Table 10:** Error Correction Representation for the Selected ARDL(1,1,1) selected based on the Akaike Information Criterion

Regressors	Coefficient	Standard Error	T-Ratio[pop]
DRN^a^	-0.042421	0.005150	-8.236941[0.0000]
DTM^b^	0.081898	0.036768	2.227421[0.0338]
ECT_1^a^	-0.834083	0.171231	-4.871112[0.0000]

R-squared = 0.855990 Adjusted R-squared = 0.846989

^a,b^Indicates statistically significant at 1%, 5%,respectively. Dependent Variable is DYL

The temperature and the variability of the average annual rainfall have a major effect on the productivity of the sesame yield, as the error correction model demonstrates. According to this finding, the coefficient of error terms (ECMt-1) in the short-run test of association by error correction term has a negative and significant value. It means that when there is a shock to sesame production and its determinant connection, the departure from the long-run equilibrium level of yields in the current period is corrected by 83.41% in the following period to restore equilibrium. Another period-lag residual that was saved from the projected dynamic long-run relationship is the ECM term. The dynamic model's adjustment to restore equilibrium is measured by N *CMt*-1*t*-1; it appears negatively and is statistically significant at the 5% level, guaranteeing the long-run equilibrium may be reached. According to [58], there is additional evidence of a stable long-term link in the form of a highly substantial error correction term. In fact, it has maintained that a more effective method of establishing cointegration is to test the importance of *ECMt*-1, which is meant to carry out a negative coefficient. Additionally, the results show that temperature and rainfall have a highly significant and respective impact on the production of sesame.This finding line with Empirical analysis of climate change factors affecting cereal yield in Turkey [59], determinants of agricultural output in Ethiopia [60, 61].

The general short-run estimated model for ARDL (1,1,1) model of sesame yield is as:

$$\Delta\mathrm{YL}=11.547+0.166\Delta{YL}_{t-1}-0.042\mathrm{RN}+0.014{RN}_{t-1}+0.082\mathrm{TM}-0106{\mathrm{TM}}_{\mathrm t-1}+0.834{ECT}_{t-1}$$

### Model stability and diagnostic test

#### Diagnostic test

Table 11 indicates that the long-run ARDL model estimated in this study passes all the diagnostic tests. This is because the *p*-value associated with both the LM version and the F version of the statistic was unable to reject the null hypothesis specified for each test. The estimated ARDL model revealed that it passed the serial correlation, normal test, heteroskedasticity, and Ramsey RESET tests. The error terms were uncorrelated, normally distributed, with the same variance, and the model was not miss-specified. Thus, they were satisfactory for the ARD model.

**Table 11:** Diagnostic test for the long-run ARDL (1, 1, 1)

ARDL(1,1,1) model for sesame yield AS the dependent variable		
Test statistic	LM version	F version
i: Serial correlation	CHSQ(2) = 3.414105[0.1814]	F(2,270 = 1.459209[0.2502]
ii: Functional form	CHSQ(2) = 5.622017[0.0601]	F(2,27) = 2.352366 [0.1143]
iii: Normality	CHSQ(2) = 1.340686[0.511532]	Not applicable
v: Heteroskedasticity	CHSQ(5) = 5.753997 [0.3309]	F(5,29) = 1.141119 [0.3614]

i: Lagrange multiplier test of residual serial correlation

ii: Ramsey’s REST test using the sequence of the fitted values

iii: Based on a test of Skewness and residuals from a Histogram table

v: Based on the regression of squared residuals on squared fitted values

#### Model stability the CUMSUM and CUSUMSQ test

**Fig. 4:**
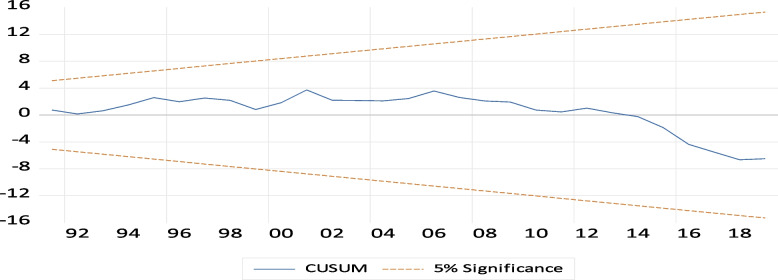
Testing parameter stability using CUSUM

**Fig. 5:**
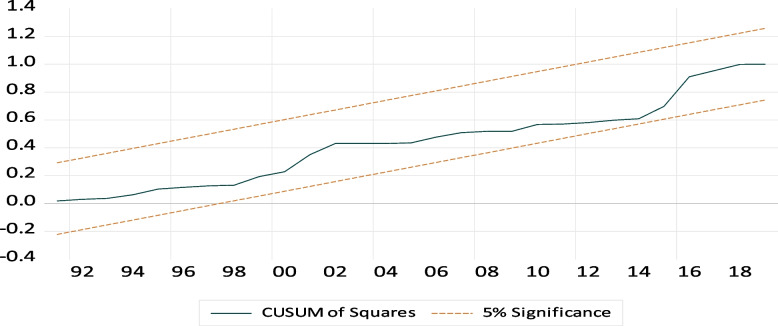
Testing parameter stability using CUSUMSQ

From the Figs. 4 and 5 two graphs, the plot of CUSUM and CUSUMSQ tests did not cross the critical limits. So, Its implies that long-run estimates are stable and there is no structural break. Testing of parameter stability using CUSUM and CUSUMSQ plot as follow:

### Structural analysis

#### Impulse Response Function (IRF)

**Fig. 6:**
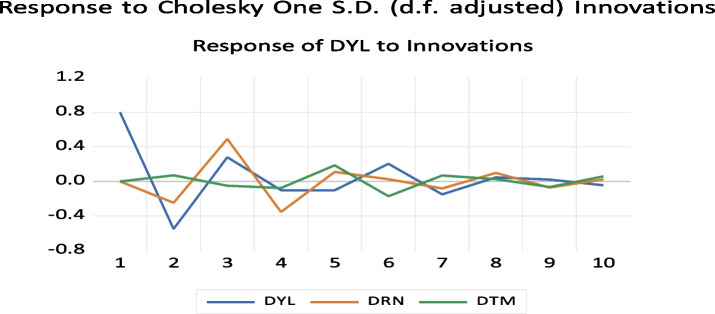
Graph of impulse response function of sesame yield

The time horizon or shock duration is indicated by the x-axis in Fig. 6, and the direction and intensity of the impulse or the percentage change in the dependent variable from its baseline level is indicated by the y-axis. There may be impulse response functions in our situation. These impulse response functions' combined graphs were used with the DRN and DTM Cholesky orderings, respectively. The reactions of DYL, DTM, and DRN to Cholesky's one standard deviation innovation in DYL are displayed in Fig. 6. The outcome suggests that advances in sesame yield have a beneficial effect on temperature. It also indicates that temperature influences sesame yield in a favorable way. It first shows a decrease as the value approaches 0.6, and then stabilizes at about the 8-year mark. Furthermore, advances in sesame yield have a detrimental effect on rainfall.

**Fig. 7:**
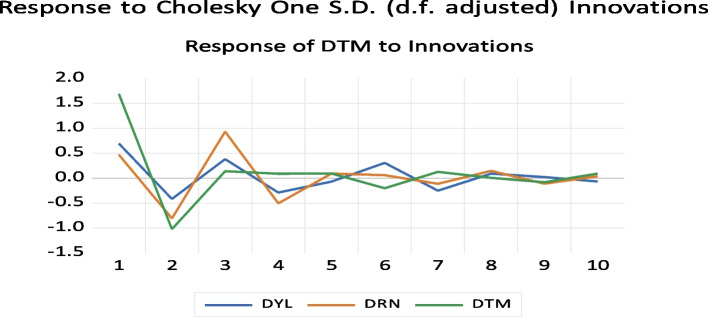
The impulse response to Cholesky one S.D innovations DTM graph

**Fig. 8:**
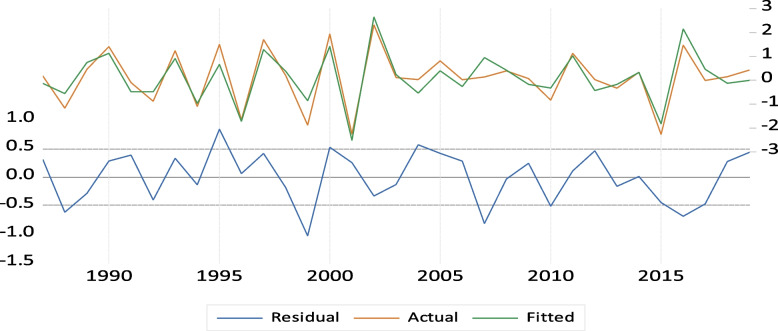
Actual, fitted and residual plot of sesame

The results of Figs. 7 and 8 show the responses of DYL, DTM, and DRN to Cholesky's one standard deviation innovation in DTM. The result indicates temperature innovations have a negative impact on rainfall. This implies that rainfall has negative effects on temperature response to Cholesky one S.D. (d.f. adjusted) novations.

#### Variance decomposition analysis

Sesame yield's variance decomposition study result reveals that, at the first horizon, its own shock (innovation), temperature, and rainfall amount of 100%, 0%, and 0%, respectively, account for the variation in yield amount. Its production innovations account for 77.51% of the variability in yield quantity fluctuations in the second year; rainfall and temperature innovations account for 21.69% and 0.80% of the variability, respectively. As the contribution of yield shocks declines, the proportion drastically drops, and temperature and rainfall shocks increase.

The variance decomposition of temperature and rainfall, respectively, reveals that rainfall in the first horizon is explained by 32.69% of its own innovation, yield accounts for 67.31% of the remaining variance, and temperature, rainfall, and yield each account for 46.62%, 0.69%, and 52.69% of its own innovation, respectively, in the second horizon. The yield and its own shock innovation are proportionately and severely reduced from the first horizon (17.84%) and 82.11%, according to the temperature variance decomposition, whereas rainfall increased from the 0.064% first-period shock.

### Forecast

#### Evaluation of forecasting accuracy

All estimated models are sufficient to describe the series, according to Table 12 findings. The sesame yield series revealed tiny mean absolute percentage error values and Thiel (U) statistics, indicating little variations between the predicted and actual values. That is, n-step forecasting can be done with the models' predictive power.

**Table 12:** Forecasting accuracy statistics

Accuracy measurement	Endogenous variable
	Yield of sesame
Root mean square error	0.42
Mean absolute error	0.35
Mean absolute percentage error	7.97
Thiel inequality coefficient	0.04
Bias proportion	0.00
Variance proportion	0.02

Using the ARDL model (1, 1, 1) for DYL is recommended based on the predicting accuracy table above, as it measures the optimal expression. This indicates that all of these values the variance percentage (0.02), bias proportion (0.00), Thiel inequality coefficient (0.04), mean absolute error (0.35), and root mean square error (0.42) are minimal. Therefore, we can conclude that there is a similarity or approach between the predicted value and the actual values of Sesame yields.

#### Out of sample forecasting analysis

Based on Table 13, which presents the annual predicted value of sesame yields, future predicted values of yield are increasing from 6.98 in 2020 to 7.48 in 2024 per year.

**Table 13:** Out of sample forecasting analysis

Year	2020	2021	2022	2023	2024
Sesame yield in (Qt/hat)	6.98	7.09	7.21	7.34	7.48

## Conclusions and policy recomandation

### Conclussion

The impact of climate change on sesame yield in North Gondar, North Ethiopia, was examined between 1987 and 2023 years using a utoregressive distributed lag (ARDL) time series model. Sesame yields, rainfall, and temperature are all cointegrated, with both short- and long-term interactions. In both the long- and short-run bound tests, there is a significant association between sesame yields and the explanatory variables (rainfall and temperature). The long-run bound test demonstrated that temperature and rainfall had a significant impact on sesame productivity.Temperature has a beneficial impact on advanced sesame yield. This suggests that temperature has a positive impact on sesame output. Rainfall reduced sesamen output in both short- and long-run testing. As rain falls, sesame production decreases. Very similar. The ARDL data confirm that temperature and rainfall have a considerable impact on sesame productivity. Temperature had a significant positive effect on sesame output, whereas rainfall had a detrimental effect in North Gondar, Ethiopia. The coefficient of error terms, on the other hand, are expected to compensate for the present period's divergence from the long-run equilibrium level of sesame yields, as the short-run test of association by error correction term has a negative and substantial value.

### Policy recommendation

First, provide insight into key policy instruments, such as the need to reconsider present government approaches to climate change in Ethiopia's North Gonder zone. Second, in order to reverse the decline in sesame yield in the North Gondar Zone due to climate change, government officials, policymakers,researchers and development planners should prioritize the deployment of novel technologies that respond to changing climate patterns. It is vital to create a modern farming policy that will increase sesame production. By doing so, the agricultural sector and other organizations that provide agricultural inputs will expand, encouraging actual investments in farming practices to increase sesame output in the country and region.

## Limitation of study

This study's analysis has only examined the relationship between sesame yields and meteorological factors like temperature and rainfall. The use of modern inputs in sesame production, pesticide use, soil fertility, agricultural methods and systems, and other factors not included in this more thorough study are ways of overcoming this limitation.

## Data Availability

No datasets were generated or analysed during the current study.

## Abbreviations

GDP: Gross Domestic Product
GCMS: Global Circulation Model
ARMA: Auto-Regressive Moving Average
VAR: Vector Auto-Regressive
AML: Agrometheorological
ADF: Augmented Dickey-Fuller
FEVD: Forecast error Variance decomposition
VEC: Vector error Correlation
ACF: Autocorrelation function
PACF: Partial Auto Correlation function
AR: Auto-Regressive
AIC: Akaike information criteria
BIC: Bayesian information criteria
HQIC: Hannan-Quinn information criteria
ME: Meanerror
MAE: Mean absolute error
RMSE: Root Mean squared error
MAPE: Mean Absolute percentage error
MPE: Mean Percentage error
LM: Lagrage Multiplier
JB: Jarque-Bera
STAR: Space-time autoregressive
SVAR: Structural Vector Autoregressive
